# Assessment of risk factors for surgical site infection following tibial plateau levelling osteotomy: a systematic review and meta‐analysis

**DOI:** 10.1111/jsap.70133

**Published:** 2026-04-08

**Authors:** S. Cavalli, F. Aragosa, C. Montano, C. Caterino, G. Della Valle, G. Fatone

**Affiliations:** ^1^ Department of Veterinary Medicine and Animal Production University of Naples Federico II Naples Italy; ^2^ Department of Veterinary Medical Sciences University of Bologna Ozzano dell'Emilia Italy

## Abstract

The aim of this systematic review was to critically assess the available evidence regarding risk factors associated with surgical site infections following tibial plateau levelling osteotomy. This study aims to identify clinically relevant determinants of surgical site infection following tibial plateau levelling osteotomy and inform future preventive strategies. This systematic review was conducted in accordance with Preferred Reporting Items for Systematic Reviews and Meta‐Analyses 2020. The review question was structured using the PECOS framework. Searches were performed in PubMed, Web of Science and Scopus without date restrictions, including all studies indexed up to June 2025. The search strategy combined terms related to surgical site infection, tibial plateau levelling osteotomy and dogs. Risk of bias was assessed across predefined methodological domains. The literature search yielded 436 results, and a total of 11 papers met the inclusion criteria. The study designs included two prospective clinical trials, one double‐blinded randomised controlled trial and eight retrospective case series. Several risk factors for postoperative surgical site infection were investigated across the studies, with increased body weight being the most frequently identified. Most of the studies presented a high risk of bias in the domains of allocation concealment, blinding of participants and personnel and blinding of outcome assessment. In contrast, incomplete outcome data and selective reporting were generally assessed as low risk of bias. Knowledge of specific risk factors for surgical site infection after tibial plateau levelling osteotomy may support the clinician in tailoring antimicrobial prophylaxis and anticipating potential complications, thereby improving perioperative decision‐making and patient outcomes.

## INTRODUCTION

Surgical site infections (SSIs) are among the most common postoperative complications in small animal surgery, with different reported prevalence rates, morbidity and mortality, according to the type of procedures (Cook et al., 2010; Eugster et al., 2004; Jessen et al., 2019; Sørensen et al., 2024) and patients' condition (Cavalli et al., 2025). Tibial plateau levelling osteotomy (TPLO), one of the most performed surgical treatments for cranial cruciate ligament injuries, has been associated with a variable incidence of SSIs, ranging from 3% to 25% (Budsberg et al., 2021; Fitzpatrick & Solano, 2010; Spencer & Daye, 2018; Tuan et al., 2019). Although TPLO typically restores full limb function and enables dogs to return to normal activity (Gordon‐Evans et al., 2013; Nelson et al., 2013), SSIs following this procedure represent a clinically significant complication. They are often associated with increased costs for owners and clinics (Budsberg et al., 2021; Fitzpatrick & Solano, 2010; Nicoll et al., 2014), a prolonged hospitalisation, patients' morbidity and the risk of development of implant‐associated infections (IAIs). Management of those infections often requires prolonged antibiotic therapies, additional surgeries and implant removal (McDougall et al., 2021; Stine et al., 2018).

Given those concerns, several studies (Aiken et al., 2015; Atwood et al., 2015; Brown et al., 2016; Clark et al., 2020; Filippo et al., 2025; Fitzpatrick & Solano, 2010; Frey et al., 2010; Gatineau et al., 2011; Lopez et al., 2018; Nazarali et al., 2014; Solano et al., 2015; Spencer & Daye, 2018; Tuan et al., 2019) have investigated the efficacy of peri‐ and postoperative surgical antimicrobial prophylaxis (SAP) in preventing SSIs following TPLO. A systematic review by Budsberg et al. (2021) synthesised part of this evidence and found little support for the use of postoperative antibiotics to reduce the risk of surgical site infections in dogs after TPLO, as the majority of studies providing this evidence were of low level of evidence, limiting their value for clinicians (Budsberg et al., 2021). However, it is well recognised that the use of antimicrobials contributes to the development of antimicrobial resistance (AMR), a major global health challenge. In this context, authors have also investigated novel non‐antibiotic techniques to reduce the incidence of SSIs following TPLO, such as triclosan‐impregnated suture materials, photo‐biomodulation therapy and silver‐coated implants (Chavez et al., 2024; Engel et al., 2024; Etter et al., 2013). The high complication rate has also prompted the exploration of machine‐learning models to predict postoperative complications in TPLO (Low et al., 2025).

Despite the considerable number of clinical studies addressing SSIs following TPLO, the specific risk factors remain poorly characterised. To our knowledge, no previous systematic review has focused on evaluating those risk factors. The aim of this systematic review was to critically assess the available evidence regarding risk factors associated with SSIs following TPLO. Furthermore, to strengthen the analysis and provide a quantitative estimate, a meta‐analysis of the available data was performed. Evidence suggests that a detailed assessment of the multifactorial nature of risk factors may contribute to improved surgical outcomes (Duran et al., 2024; Marzoug et al., 2023). This study aims to identify clinically relevant determinants of SSI following TPLO and inform future preventive strategies.

## MATERIALS AND METHODS

### Search strategy

This systematic review was conducted and reported in accordance with the Preferred Reporting Items for Systematic Reviews and Meta‐Analyses (PRISMA 2020) statement (Page et al., 2021). The review protocol was registered in Open Science Framework (OSF) (registration number: 10.17605/OSF.IO/9ZX5Q). The review question was structured according to the PECOS framework: Population, dogs undergoing tibial plateau levelling osteotomy (TPLO); Exposure, risk factors potentially associated with postoperative SSI; Comparator, dogs not exposed to the factor of interest or exposed to a different level of the investigated variable, where applicable; Outcome, occurrence of postoperative SSI; Study design, randomised clinical trials and observational studies (prospective or retrospective) evaluating risk factors for SSI after TPLO.

The search for relevant publications was not restricted by publication date and included all studies indexed in the selected databases up to June 2025. The databases used were PubMed, Web of Science and Scopus. The literature search was conducted independently by three authors (SC, CC and FA), and any disagreements were resolved through discussion or, when necessary, by consultation with two additional authors (GDV and GF). The following search strategy was used: (“surgical site infection” OR “SSI” OR “postoperative infection”) AND (“tibial plateau levelling osteotomy” OR “TPLO”). Searches were performed in the title, abstract and keywords fields of each database. In addition, the reference lists of relevant studies were manually screened to identify further eligible articles.

### Inclusion criteria

Inclusion criteria involved the presence of relevant terms such as “SSI”, “Surgical site infection”, “Postoperative infection”, “TPLO”, “tibial plateau levelling osteotomy”, in the full manuscript, abstract, title and keywords of publications indexed by web search engines that provide access to full‐text articles (PubMed, Web of Science and Scopus).

We included randomised controlled trials (RCTs), prospective cohort studies, retrospective studies, systematic reviews, scoping reviews and case series involving a minimum of 15 dogs that underwent TPLO. Eligible studies were required to specifically evaluate the risk factors associated with SSIs following TPLO.

Only full‐text articles published in peer‐reviewed journals and written in English were considered.

### Exclusion criteria

All papers published in languages other than English were excluded. Studies focusing on osteotomies other than TPLO for the treatment of CrCL rupture were not considered. All studies involving species other than dogs were excluded. All human, cadaveric or *in vitro* studies were excluded. Studies including only a limited population by age, breed, body size or other characteristics that could themselves represent a risk factor were also excluded.

In addition, abstracts, book chapters, conference or congress proceedings and letters to the editor were excluded. Studies without specific data on the risk factors increasing SSIs following TPLO, case reports and case series involving fewer than 15 dogs were also excluded.

### Selection process

Duplicate records were removed manually. After duplicate records were removed, three authors (SC, CC and FA) independently screened the titles manually according to predefined inclusion and exclusion criteria. Any disagreements were resolved through discussion or, if necessary, by the fourth and fifth authors (GDV and GF). The publications that remained after this manual title screening were further assessed by reading the abstracts, applying the same criteria used for the title screening. Next, four researchers (SC, CC, FA and GDV) independently screened full texts to determine eligibility. Again, all discrepancies were resolved between the authors, with the availability of a fifth author (GF).

### Data extraction

Two authors (XX and XX) conducted data extraction independently. Data extracted included: first author, year of publication, journal of publication, study design, SSI incidence (percentage), SSI classification, risk factors, odds ratio (OR), surgical antimicrobial prophylaxis (SAP), sample size, follow‐up period (days), model of analysis (*e.g*. univariate and multivariate).

Data extracted from the included studies were entered into a standardised spreadsheet using Microsoft Excel (Microsoft Corporation, Redmond, WA, USA). When relevant information was missing or unclear, other attempts were made to retrieve it from the original text, tables or supplementary material; no imputation of missing data was performed.

### Classification

The study design was determined for each article included in the review. Systematic reviews of homogeneous randomised controlled trials were considered the highest level of evidence, followed by blinded randomised clinical trials (bRCTs), non‐blinded randomised clinical trials (nbRCTs), non‐randomised clinical trials (NRCTs), uncontrolled clinical trials (UCTs) and case series (CSs). Case reports (CRs) and expert opinion were considered to provide the lowest level of evidence and were excluded (Straus et al., 2011). Papers that met the inclusion criteria were classified according to the Grading of Recommendations, Assessment, Development and Evaluations (GRADE) (Atkins et al., 2004) system for evaluating clinical evidence and according to the Oxford Centre for Evidence‐Based Medicine (CEBM) – Levels of Evidence (Oxford Centre for Evidence‐Based Medicine, 2009). The GRADE system mandates that a study initially be given one of three grades: 1 = high, 2 = low or 3 = very low. High‐grade studies are systematic reviews and bRCTs, low‐grade studies are observational and very low studies are considered any other type of evidence. After initial grade assessment, the grade can increase or decrease following the GRADE system criteria for assigning a grade of evidence (Atkins et al., 2004). The Oxford CEBM system classifies evidence into five levels, from 1 (highest) to 5 (lowest). Level 1a corresponds to a systematic review of homogeneous, blind randomised controlled trials, while level 5 represents expert opinion without explicit critical appraisal.

### Risk of bias

The risk of bias of the selected studies was assessed using the Cochrane Collaboration Risk of Bias tool, evaluating the domains of sequence generation, allocation concealment, blinding, incomplete outcome data, selective reporting and other potential sources of bias. The results were presented according to the PRISMA guidelines (Moher et al., 2009), and trials were assigned as having a high, low or unclear risk of bias.

### Meta‐analysis

A quantitative analysis of the available literature on postoperative infections following TPLO was conducted using the R platform with OpenMeta[Analyst] (Wallace et al., 2012). To estimate the pooled effect, a random‐effects meta‐analysis model was applied. The results were illustrated using a forest plot. All statistical tests were two‐tailed, with the level of significance set at *P* < .05. The Mantel–Haenszel (M–H) method was used to determine the weight of each study. Heterogeneity across studies was assessed using the *I* ^2^ statistic, with values of approximately 25%, 50% and 75% representing low, moderate and high heterogeneity, respectively.

A completed PRISMA 2020 checklist is provided as Supplementary Material.

## RESULTS

### Study selection

**FIG 1:**
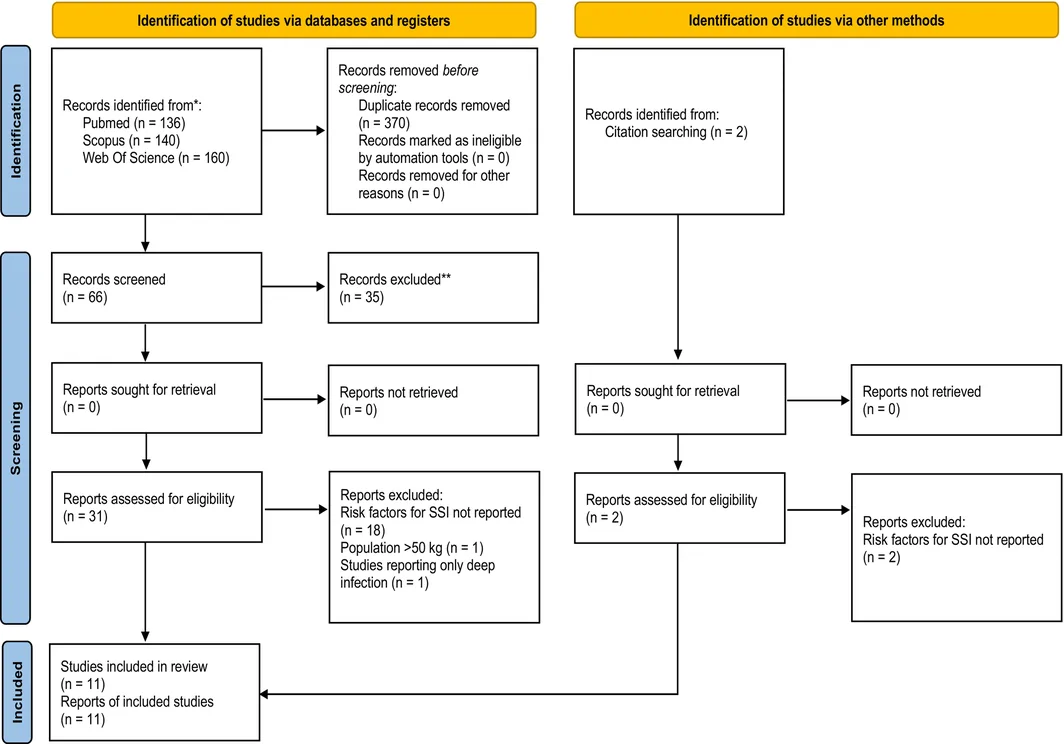
Prisma 2020 flow diagram for a new systematic review, which included databases, registers and other sources (Page et al., 2021). *Consider, if feasible to do so, reporting the number of records identified from each database or register searched (rather than the total number across all databases/registers). **If automation tools were used, indicate how many records were excluded by a human and how many were excluded by automation tools.

The literature search yielded 436 results, as shown in Fig 1; two additional records were extracted through other sources. After removing duplicates, 68 papers were retained. After title and abstract screening, 33 potential papers were retained for full‐text evaluation. Twenty‐two studies did not meet the inclusion criteria (Fig 1) and were excluded from the study. A total of 11 studies (Atwood et al., 2015; Clark et al., 2020; Fitzpatrick & Solano, 2010; Giannetto & Aktay, 2019; Hagen et al., 2020; Husi et al., 2023; Lopez et al., 2018; Nazarali et al., 2014, 2015; Sanders et al., 2024; Spencer & Daye, 2018) met the inclusion criteria and were included in the systematic review.

Of the 22 studies of literature search that did not meet the inclusion criteria, 18 were excluded because they did not report any data on risk factors for SSI following TPLO (Aryazand et al., 2023; Brown et al., 2016; Chavez et al., 2024; Chiu et al., 2019; Engel et al., 2024; Etter et al., 2013; Ferrari et al., 2024; Filippo et al., 2025; Mann & Barr, 2025; Nicoll et al., 2014; Oberhaus & McFadden, 2023; Pagès et al., 2022; Peress et al., 2021; Savicky et al., 2013; Stine et al., 2018; Throckmorton et al., 2009; Tuan et al., 2019; Watson et al., 2019). One study (Solano et al., 2015) was excluded because it included only dogs weighing more than 50 kg, without distinguishing whether they were overweight or of normal weight, thereby considering only giant breeds; as both breed and overweight are potential risk factors, this selection could influence the study population. Another study (McDougall et al., 2021) was excluded because it addressed only deep SSIs, thereby omitting cases of superficial or organ/space SSIs and the associated risk factors. In addition, two studies retrieved from the reference lists of eligible articles were assessed in full text but subsequently excluded because they did not provide any data on SSI (Bergh & Peirone, 2012; Coletti et al., 2014).

### Data extraction

A total of 11 papers published between 2010 and 2023 met the inclusion criteria. Seven were published in *Veterinary Surgery* (*VetSurg*), two in the *Journal of the American Veterinary Medical Association* (*JAVMA*), one in the *Canadian Veterinary Journal* (*Can Vet J*), one in *Veterinary and Comparative Orthopaedics and Traumatology* (*VCOT*) and one in *Frontiers in Veterinary Science* (Frontiers). The study designs included two prospective clinical trials, one double‐blinded randomised controlled trial (dbRCT) and eight retrospective case series.

The sample sizes varied between 134 and 1422 stifles, with the incidence of SSI reported to range from 2.3% to 25%. Only one study classified infections according to CDC classification, identifying superficial, deep and organ/space SSIs; another study classified the SSIs into major and minor, while the remaining papers did not provide such classification.

Several risk factors for postoperative SSI were investigated across the studies. Spencer and Daye (2018) reported body weight as significantly associated with SSI occurrence. Nazarali et al. (2015) identified *methicillin‐resistant Staphylococcus pseudintermedius* (MRSP) carriage as a risk factor. Hagen et al. (2020) found that both younger (<6 years) and older (>12 years) dogs had an increased risk of infection. Atwood et al. (2015) also highlighted body weight as a contributing factor. Clark et al. (2020) demonstrated that longer duration of surgery, bilateral procedures, left‐sided TPLO, the primary surgeon's expertise, postoperative doxycycline use *versus* other protocols and body weight were associated with higher SSI risk. Sanders et al. (2024) confirmed body weight as a risk factor and additionally reported increased risk in dogs undergoing bilateral procedures in a single session. Nazarali et al. (2014) identified longer anaesthesia time as a relevant variable. Fitzpatrick and Solano (2010) described male intact status and body weight as significant risk factors. Giannetto and Aktay (2019) also confirmed the role of body weight. Lopez et al. (2018) reported German shepherd dog breed, concurrent meniscectomy and limited surgeon experience as significant risk factors. Finally, Husi et al. (2023) demonstrated an increased risk in German shepherd dogs and in those with a history of contralateral TPLO.

Most studies used cefazolin as SAP, although alternative drugs (e.g., cefuroxime) or variable protocols were reported. Minimum follow‐up ranged from 10 days to 1 year; three papers did not report the minimum follow‐up. All included studies assessed risk factors through multivariate analysis.

The data extracted from each study included in the review are summarised in Table 1.

**Table 1:** The summary of data extracted from each study included in the review

First author	Year	Journal	Study design	SSI (%)	SSI class	Risk factors	OR	SAP	Sample size	Follow‐up
Spencer	2018	VetSurg	dbRCT	14.2	Not present	Body weight (*P* = .003)	1.047	Cefazolin 22 mg/kg	134 stifles	Min 56 days
Nazarali	2015	JAVMA	Prospective clinical trial	6.2	Not present	MRSP carriage (*P* < .001)	6.72	Various	549 stifles	30 to 365 days
Hagen	2020	VetSurg	Retrospective case series	11	Not present	Age <6 and >12 years (*P* =.008)	1.65	Cefazolin 25 mg/kg	659 stifle (*n* = 541)	Min 1 follow‐up
Atwood	2015	Can Vet J	Retrospective case series	10.8	Not present	Body weight (*P* =.01)	1.04	Cefazolin 22 mg/kg	306 stfles	Not reported
Clark	2020	VetSurg	Retrospective case series	25	50 superficial 29 deep	Duration of surgery (*P* = .036) Bilateral TPLO (*P* = .049) Left‐side TPLO (*P* = .036) Primary surgeon (*P* = .006) Doxycicline as post SAP (*P* = .034) Body weight (*P* = .008)	Not present	Various	208 stifles	Min 365 days
Sanders	2023	VetSurg	Retrospective case series	11	Not present	Bilateral TPLO (*P* = .004) Body weight (*P* = .001)	2.5 1.11	Cefazolin	1422 stifles	Min 365 days
Nazarali	2014	VetSurg	Retrospective case series	13.3	Not present	Anaesthesia time (*P* = .01)	1.009	Cefazolin	226 stifles	Not reported
Fitzpatrick	2010	VetSurg	Retrospective case series	6.6	Not present	Being intact male (*P* = .05) Body weight (*P* = .01)	1.85 1.02	Cefuroxime	1146 stifles	Min 180 days
Giannetto	2019	VCOT	Prospective clincal trial	2.3	Not present	Body weight (*P* < .001)	1.03	Cefazolin 22 mg/kg	437 stifles	Min 90 days
Lopez	2018	JAVMA	Retrospective case series	8.4	Not present	German shepard (*P* < .001) Meniscectomy (*P* = .02) Surgeon who performed <20 TPLO (*P* = .02)	9.72 2.49 2.96	Cefazolin >20 mg/kg	405 stifles	Not reported
Husi	2023	Frontiers	Retrospective case series	8.5	52 major 13 minor	Previous TPLO (*P* = .02) Germand shepard (*P* = .035)	2.01 4.44	Various	769 stifles	10 to 3029 days

Min Minimum

### Classification

Each study included in this review was classified for level of evidence as reported in Table 2, following the GRADE and Oxford CBEM systems.

**Table 2:** Level of evidence for each study following GRADE and Oxford CBEM systems

First author	GRADE	Oxford CBEM
Spencer	High	1b
Nazarali	Low	2b
Hagen	Low	4
Atwood	Very low	4
Clark	Low	4
Sanders	Low	4
Nazarali	Very low	4
Fitzpatrick	Low	4
Giannetto	Low	2b
Lopez	Low	4
Husi	Low	4

### Risk of bias

**FIG 2:**
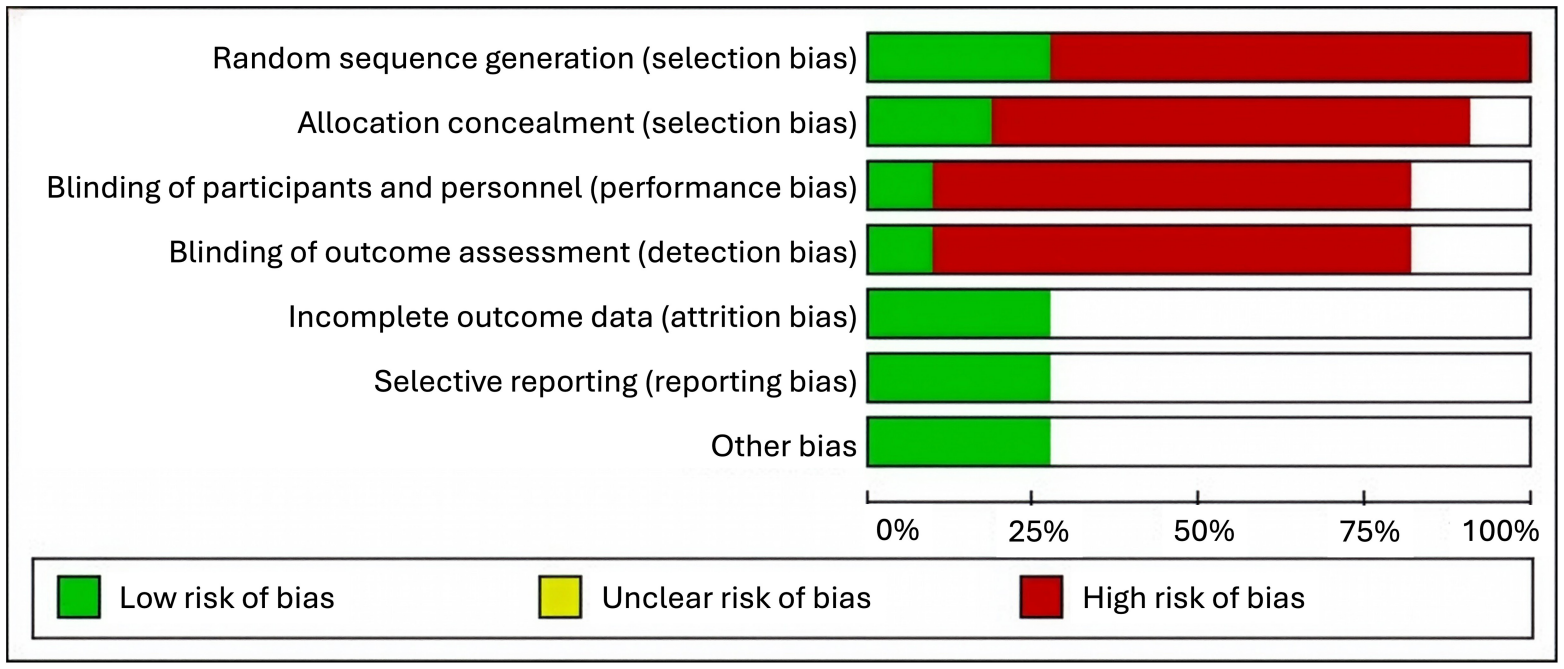
Risk of bias summary – review authors' judgements about each risk of bias domain across all included studies.

**FIG 3:**
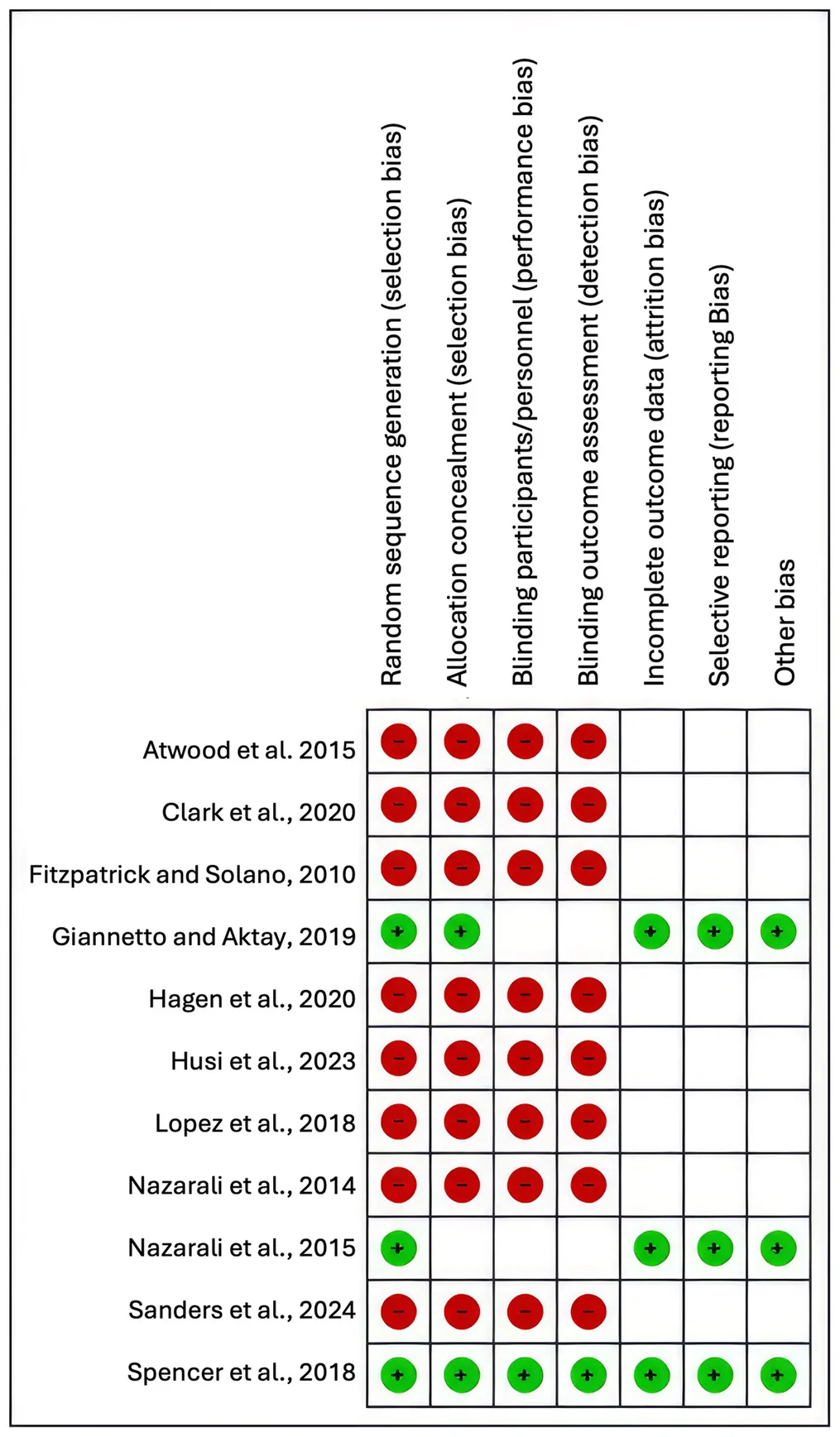
Risk of bias for individual studies – review authors' judgements about each risk of bias domain for each included study.

The overall risk of bias of the studies included in the systematic review and meta‐analysis is summarised in Figs 2 and 3. Most of the studies presented a high risk of bias in the domains of allocation concealment, blinding of participants and personnel and blinding of outcome assessment. In contrast, incomplete outcome data and selective reporting were generally assessed as low risk of bias. Random sequence generation was often unclear or inadequately reported.

### Quantitative analysis

**FIG 4:**
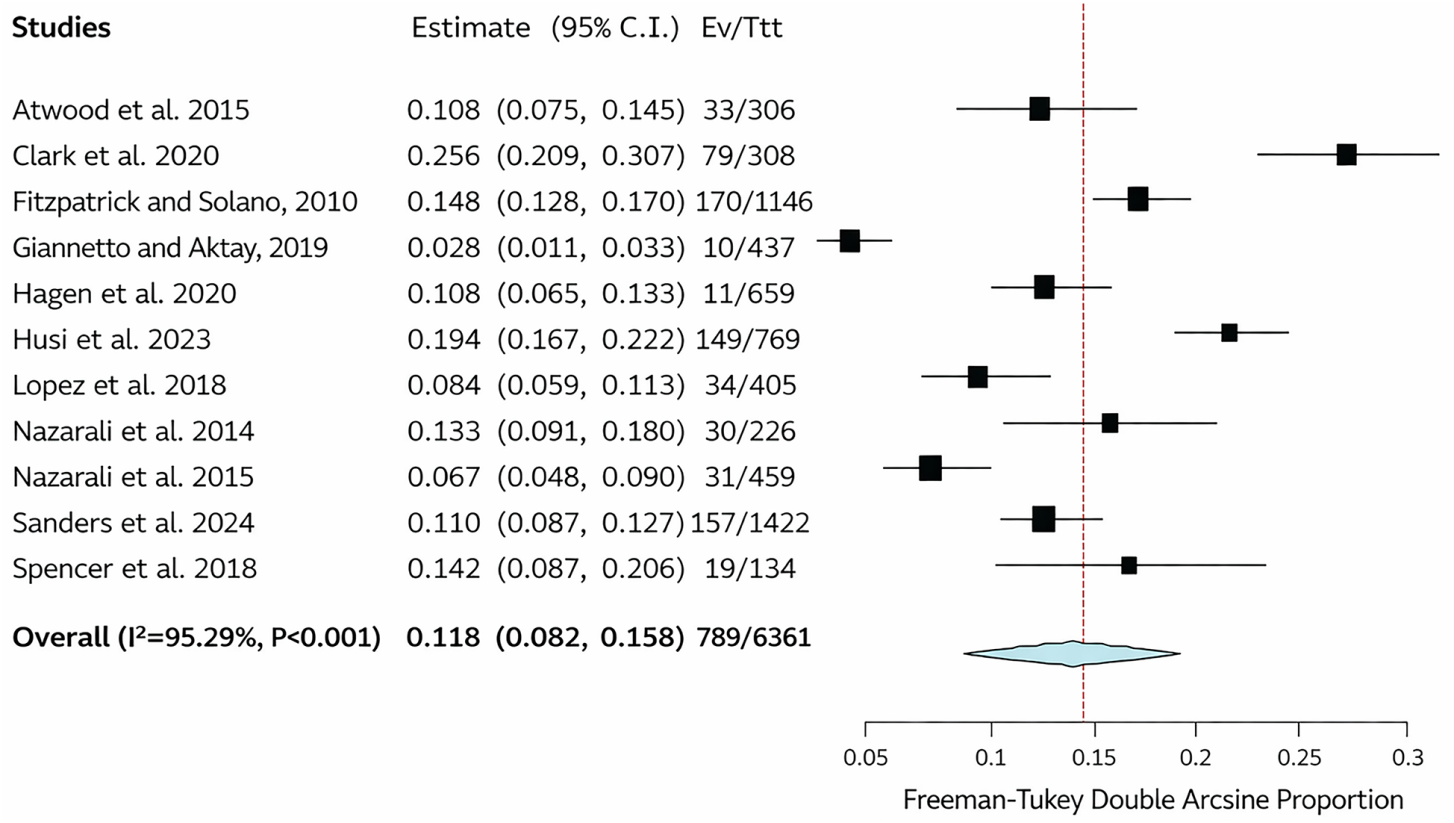
Forest plot of all the studies included in the meta‐analysis.

Results of the overall meta‐analysis are summarised in the forest plot in Fig 4. Pooling data from the 11 included studies (6361 cases and 789 events), the estimated overall proportion of SSI was 11.8% (95% CI: 8.2% to 15.8%). However, heterogeneity was high (*I* ^2^ = 95.29%, *P* < .001), indicating considerable variability among studies.

## DISCUSSION

This is the first systematic review to evaluate the risk factors for SSIs following TPLO, one of the most performed procedures in small animal orthopaedic surgery. Reported SSI rates remain variable and can reach 25% (Clark et al., 2020), the highest incidence among cruciate stabilisation techniques. Given this high frequency, the authors propose that risk factor assessment should become part of the preoperative evaluation, as it may assist surgeons in antimicrobial decision‐making and anticipating potential complications.

Among the risk factors analysed in the present study, body weight was the most consistently associated with SSI after TPLO. Significant associations were reported in different studies (Atwood et al., 2015; Clark et al., 2020; Fitzpatrick & Solano, 2010; Giannetto & Aktay, 2019; Sanders et al., 2024; Spencer & Daye, 2018) with odds ratio (OR) ranging from 1.02 to 1.11. Increased body weight has also been identified as a risk factor for SSI after lateral fabellar suture stabilisation (Cox et al., 2020). These findings concern absolute body weight and not obesity. In human orthopaedic surgery, a systematic review on anterior cruciate ligament reconstruction and one on SSI after clean orthopaedic surgery (Liu et al., 2025) similarly highlighted obesity as a risk factor of SSI (Zhao et al., 2023). Differentiating between absolute body weight and obesity in small animals would therefore be of relevance to better understand whether the increased risk is primarily related to mechanical load or to metabolic or systemic factors.

The German shepherd dog breed was another risk factor identified in different studies (Husi et al., 2023; Lopez et al., 2018). The higher predisposition of German shepherd dogs to dermatological conditions (Bizikova et al., 2015; Jaeger et al., 2010; Picco et al., 2008; Wilhem et al., 2011), like atopic or seborrheic dermatitis, could partly explain their increased susceptibility to SSIs, but further studies are needed to confirm this hypothesis.

Several additional risk factors have been reported, although only in single studies. Those included age (<6 years or >12 years), intact male status, MRSP carriage (Nazarali et al., 2015), bilateral procedures performed in a single session (Sanders et al., 2024), concurrent meniscectomy (Lopez et al., 2018), surgeon experience (Lopez et al., 2018) and prolonged anaesthesia/surgical time (Clark et al., 2020; Nazarali et al., 2014) were identified as risk factors for SSIs after TPLO. However, body weight was the only factor supported by studies with the highest level of evidence and comparatively lower risk of bias (Giannetto & Aktay, 2019; Spencer & Daye, 2018). All other factors, except MRSP carriage reported by Nazarali et al. (2014), were reported exclusively in retrospective studies, and presented a high risk of bias and a low LoE. For this reason, while body weight can be considered a consistent and reliable risk factor, the role of the other factors cannot be confirmed and no meaningful conclusions can be drawn.

The review has several limitations. The included studies showed high variability in study design, definitions of risk factors and outcome assessment. For instance, some studies analysed anaesthesia time or ASA, while others did not; several excluded patients' comorbidities or endocrinopathies, and follow‐up duration was inconsistently reported. Only two studies provided a follow‐up of at least 12 months, while three did not report the follow‐up time. Moreover, 8 of the 11 included papers were retrospective studies, which introduces bias and weakens the strength of the conclusions.

The quantitative analysis also highlighted the high variability between studies. The present meta‐analysis showed a high level of heterogeneity (*I* ^2^ = 95.29%), which considerably limits the robustness of the findings. This emphasises the need for future research, including a larger number of studies with more homogeneous designs and populations. Moreover, it was not possible to conduct subgroup meta‐analyses on specific risk factors, such as body weight, German shepherd dog breed, surgical time or concurrent procedures, since fewer studies consistently investigated each of them. This limitation prevented the authors from reducing the source of heterogeneity and from providing a more precise estimate of the influence of these variables on SSI occurrence.

The authors suggest that future research should focus on prospective studies with standardised definitions and reporting. Key data to be collected should include age, sex, breed, body weight with categorisation (e.g. normal weight and obese), ASA status, SAP, surgical time, anaesthesia time, whether both TPLO procedures are performed in the same session, wound classification, SSI classification according to CDC guidelines, and a minimum follow‐up of at least 6 months.

This systematic review and meta‐analysis provide the first comprehensive evaluation of risk factors for SSI following TPLO. Despite the high risk of bias in the quantitative analysis, body weight appears to play a significant role in the development of postoperative SSIs. Other factors, including breed (German shepherd), age, sex and surgical variables, were identified in some studies but remain inconclusive due to low‐quality evidence.

Overall, the considerable heterogeneity among studies highlighted in the present meta‐analysis prevents definitive conclusions. Further research is needed to standardise study designs and minimise bias to more accurately identify SSI risk factors and develop effective preventive strategies.

## Author contributions

**S. Cavalli:** Conceptualization; methodology; data curation; investigation; formal analysis; writing – original draft; writing – review and editing. **F. Aragosa:** Data curation; investigation; validation; visualization; writing‐original draft; writing‐review and editing. **C. Montano:** Formal analysis; writing – original draft; writing – review and editing. **C. Caterino:** Conceptualization; data curation; investigation; validation; visualization; writing – original draft; writing – review and editing. **G. Della Valle:** Data curation; investigation; formal analysis; writing – original draft; writing – review and editing. **G. Fatone:** Conceptualization; methodology; validation; supervision; visualization; project administration; writing – original draft; writing – review and editing.

## Conflict of interest

No conflicts of interest have been declared.

## Supporting information


**Data S1.** PRISMA 2020 checklist

## Data Availability

The data that support the findings of this study are available from the corresponding author upon reasonable request.
